# Addressing animal methods bias through government-led initiatives

**DOI:** 10.3389/ftox.2026.1761759

**Published:** 2026-01-29

**Authors:** Jessica S. Kopew, Merel Ritskes-Hoitinga, Mikalah Singer, Emily R. Trunnell, Gabby Vidaurre, Catharine E. Krebs

**Affiliations:** 1 Physicians Committee for Responsible Medicine, Washington, DC, United States; 2 University of Utrecht, Utrecht, Netherlands; 3 Aarhus University, Aarhus, Denmark; 4 People for the Ethical Treatment of Animals (PETA), Washington, DC, United States

**Keywords:** animal methods bias, animal-free research, new approach methodologies, peer review bias, research policy

## Abstract

Peer review aims to ensure that high-quality research is funded, conducted, and disseminated. However, peer review bias limits this process by violating impartiality, allowing factors other than merit to influence reviewers’ judgment. Animal methods bias is a peer review bias characterized by a preference for animal-based methods or lack of expertise to properly evaluate nonanimal methods, affecting the fair consideration of animal-free biomedical approaches. To date, most investigations into the characteristics of animal methods bias have relied on self-reported survey data and anecdotes from workshops, but available evidence shows it can impact the likelihood of obtaining funding or achieving positive publication outcomes and may also lead to the unjustified use of animals because of undue pressure caused by reviewer expectations or requests. Globally, progress is advancing in the shift away from animal experimentation and towards human-based, nonanimal methods to improve translation and clinical relevance, but barriers like animal methods bias must be understood and addressed to see these efforts through and reach the full potential of new approach methodologies (NAMs). In 2025, two government-led initiatives were announced to this end, in the United States and United Kingdom. In addition to measures like expanded funding, training, and infrastructure for nonanimal methods, these initiatives also included efforts to address animal methods bias. While global recognition of animal methods bias is encouraging, the implementation and success of these measures remains to be seen, and further strategies to understand and address animal methods bias around the world and throughout the research lifecycle are needed.

## 1 Introduction

Peer review plays a central role in determining which biomedical research is supported and published, with the goal of promoting rigorous and impactful science. Yet the system is not without shortcomings. Among the challenges is the presence of biases that can shape reviewers’ evaluations, allowing considerations unrelated to scientific merit to influence decisions (Lee et al., 2012). This perspective examines one such bias, referred to as “animal methods bias,” in which animal-based approaches are favored or nonanimal methods are undervalued due to limited familiarity or confidence in assessing them (Krebs et al., 2023a; Krebs et al., 2023b; Krebs and Herrmann, 2024; Parvatam et al., 2025; Krebs et al., 2025). This dynamic can hinder the fair evaluation of emerging animal-free technologies, including complex *in vitro* models, artificial intelligence, and other such new approach methodologies (NAMs) aimed at improving biomedical translation and reducing and replacing animals. Animal methods bias appears at multiple stages of the research lifecycle, especially during the peer review of manuscripts for publication or grant applications, potentially influencing funding and publishing outcomes, and even prompting researchers to incorporate animal experiments they would not otherwise conduct, raising both ethical and scientific concerns (Krebs et al., 2023a; Krebs et al., 2023b; Krebs and Herrmann, 2024; Parvatam et al., 2025; Krebs et al., 2025).

Many governments around the world are implementing policies in support of human-based NAMs to improve disease modeling and regulatory testing, advance translation to clinical outcomes, and replace animal use. In the European Union, the Registration, Evaluation, Authorisation and Restriction of Chemicals regulation came into effect in 2007, partly aimed at promoting nonanimal methods for chemical risk assessment. ECHA (2025) Directive 2010/63/EU set a goal to phase out animal use for research and regulatory testing, and at the time of writing, the European Commission is developing a roadmap toward this goal. European Commission (2025) Similar progress has been made in Brazil to incorporate NAMs in regulatory testing (Villela and Machado, 2022). Meanwhile, the Netherlands sets a global example by investing public funds in programs like More Knowledge with Fewer Animals (ZonMw, 2024) and the new Ombion Centre for Animal-Free Biomedical Translation, which aims to build a thriving national animal-free infrastructure (Ombion, 2025). It is thus more important than ever to understand the nature of animal methods bias and how to best combat it so that it is not a hindrance to this exciting global progress.

Two new government-led initiatives in the United States and United Kingdom were announced in 2025 aimed at bolstering the development and use of nonanimal methods while replacing animals, and both included measures to address animal methods bias (U.S. National Institutes of Health, 2025; U.K. Department for Science, Innovation and Technology, 2025). This perspective examines how animal methods bias influences the peer review of grant applications and studies for publication, assesses how these new initiatives may help mitigate animal methods bias, and explores further action needed from global research agencies to address this issue amid the growing shift away from animal use in science.

## 2 Animal methods bias in the review of grant applications

Scientific research depends heavily on both public and private funding. Grant applications are typically evaluated through peer review processes that determine which projects should receive support. When reviewers have limited expertise with nonanimal methods or hold biases favoring animal-based approaches, projects using nonanimal methods may be disadvantaged compared with similar proposals involving animals. One study found that among NIH neuroscience grants funded over an 8 month period, 72% involved animal use, while only 16% and 12% relied on new NAMs and human studies, respectively (Trunnell and Roe, 2025). This imbalance may be partly explained by the composition of the review panels: the predominant expertise of reviewers was in animal-based methods (43%), compared to 33% molecular/cellular expertise and 22% human (clinical) expertise (Trunnell and Roe, 2025). This skew toward animal expertise was correlated with a higher likelihood of funding animal-related projects and a lower likelihood of supporting human-based or NAMs-focused proposals (Trunnell and Roe, 2025). These findings drive home the importance of integrating appropriate expertise in review groups.

At a “Workshop to Explore Animal Methods Bias in Biomedical Research Funding” in May 2024, a majority of attendees found animal methods bias to be a significant barrier to obtaining funding (Krebs et al., 2025). Participants shared first-hand accounts of reviewers insisting on employing animal methods to validate *in vitro* methods (even when the animal methods in question did not exist), preferring animal data over more clinically relevant patient-derived data, and rejecting NAMs-based proposals outright (Krebs et al., 2025). While some of this may be due to reviewers favoring animal-based methods, it can also be explained by their familiarity with animal-based methods or lack of awareness of human-based methods and their many translational advantages.

Further investigating researcher experiences and perspectives on this matter, another study found that over half of those surveyed believed their grant proposal evaluations were negatively influenced by the lack of animal experiments (Parvatam et al., 2025). These studies illustrate that animal methods bias can unfairly divert research funding toward animal-based science, putting pressure on researchers to add animal experiments to their proposals just to secure funding. They also point to potential measures to address this issue, like appropriate expertise in review groups, bias mitigation training, and simple educational or awareness campaigns about human-based methods.

## 3 Animal methods bias in the review of studies for publication

On the other end of the scientific funding cycle is the publication of completed research. This stage is also subject to peer review, which evaluates whether the work is appropriate for the chosen journal and meets standards of quality, impact, and scientific rigor. Here, similar issues arise due to biases held against and lack of knowledge about nonanimal methods (Krebs et al., 2023a; Krebs et al., 2023b). One study found that of the researchers surveyed, 31% had at some point added animal experiments to a manuscript solely in anticipation of peer reviewer requests for them (Krebs et al., 2023b). This same study found that 46% of respondents had been requested by reviewers to add animal experiments to an otherwise animal-free manuscript at least once (Krebs et al., 2023b). Another study found that surveyed authors chose not to comply with reviewer requests for animal experiments about three in every four instances (76%), citing reasons like a lack of feasibility, that the suggested experiment was beyond the original scope of the study, and that a nonanimal approach was provided instead (Parvatam et al., 2025).

Animal methods bias in publishing can reduce the impact of nonanimal research, delay publication, discourage researchers, and negatively impact the ability of researchers to obtain funding (Krebs et al., 2023b; Parvatam et al., 2025). As in the grant review process, these issues can put undue pressure on researchers to use animals when they do not feel it is scientifically necessary. This can also lead to the conduct of animal experiments that would not have otherwise been performed, distorting the scientific literature and interpretations about the necessity of animal use. If biases toward animal methods in publishing remain unaddressed, the acceptance, development, and use of human-based methods will be impeded.

## 4 Global policies acknowledge animal methods bias

There are increasing global calls to move away from animal use and towards human-based, nonanimal research. Recent initiatives in the US have made promising shifts towards the acceptance and use of nonanimal methods. In April 2025, the FDA announced a plan to phase out animal testing requirements for monoclonal antibodies and other drugs (U. S. Food and Drug Administration, 2025a). A few weeks later, the NIH introduced an initiative to prioritize human-based research while reducing animal use, including the establishment of a new office to coordinate NIH-wide efforts and expand funding, training, and infrastructure for nonanimal methods (U.S. National Institutes of Health, 2025). In July 2025, the NIH stated that it would no longer release funding opportunities exclusively calling for animal-based research proposals (U. S. Food and Drug Administration, 2025b).

As a part of its new human-based research initiative, the NIH announced that “grant review staff will participate in mitigation training to address any possible bias towards animal studies and integrate experts on alternative methods into study sections” (U.S. National Institutes of Health, 2025). As laid out above, the bias does indeed exist, and having appropriate expertise in review groups is crucial to ensuring human-based research proposals are reviewed fairly (Krebs et al., 2025; Trunnell and Roe, 2025). However, in addition to these measures, the agency should investigate the full extent and characteristics of animal methods bias [or facilitate external meta-researcher investigations, as the agency did for racial bias (Ginther et al., 2011)] by assessing review reports and comments during review group meetings for evidence of bias and the resulting impacts on funding rates of animal- and human-based projects. Large language models could be particularly useful for screening review reports for biased comments. Cooperation with social scientists is also essential for understanding how human behavior contributes to animal methods bias and for identifying other hurdles and opportunities that influence the shift to animal-free research [the Netherlands provides public funds for three such interdisciplinary consortia (NWO, 2021)]. Additionally, review staff *and* reviewers should participate in bias mitigation training, and they should be informed of the translational value of human-based methods—a measure already proposed by an agency advisory group tasked with articulating needs for catalyzing the development and use of NAMs (Chang, 2023). Lastly, the NIH could do more to impact animal methods bias at journals. It already provides publishing guidance to researchers and undoubtedly wields some amount of power over biomedical research publishing stakeholders (U. S. National Institutes of Health 2025).

In November 2025, the United Kingdom released a strategy for replacing animals in science, including new funding for developing and testing NAMs as well as increased training in NAMs (GOV.UK, 2025). The strategy lists barriers to the adoption of alternative methods, including “[c]oncerns about lack of support and acceptance from peers, scientific journals and regulators” and “[p]oor knowledge about the availability of alternative methods and institutional commitment to *in vivo* models,” both of which feed into bias that favors animal methods (GOV.UK, 2025). It includes several measures to help address this issue as it relates to publishing: ensuring that editorial policies incentivize the use of these methods without requiring the inclusion of additional animal experiments; providing guidance for peer reviewers on fair evaluation of nonanimal methods; and requiring metrics to track how many animal versus nonanimal studies a journal publishes to measure progress. The strategy also includes measures to address bias in funding decisions: increasing awareness and training to ensure researchers and institutions understand and adopt innovative approaches; and empowering funding panels to scrutinize animal experiments and support robust, nonanimal methods. While more comprehensive in addressing animal methods bias than any other government-led initiative to date, the strategy omits important measures to provide bias mitigation training to grant application reviewers, ensure appropriate human-based expertise among reviewers at all stages, and investigate animal methods bias characteristics and impacts on funding rates.

These initiatives show promise in shifting away from animal use in biomedical research and begin to address animal methods bias, serving as a model for how other nations might also put forth change. However, neither explicitly names “animal methods bias.” Doing so would: (1) help legitimize this phenomenon that has very real impacts on researchers who use NAMs and other nonanimal methods; (2) promote the future study of animal methods bias by meta-researchers and social scientists, which will be crucial in better understanding and characterizing it; and (3) help standardize terminology to connect diverse stakeholders and foster harmonization across disparate mitigation efforts. Further, the implementation of stated plans remains to be seen, and additional measures, as described above, will be necessary to holistically address this issue. Unless animal methods bias is addressed as broadly as possible, it will remain a barrier to broader NAMs uptake, and these initiatives and others will continue to meet resistance.

## 5 Policy recommendations to mitigate animal methods bias

To address and mitigate animal methods bias in research funding and publishing, a number of steps are needed. Below is a synthesized list of recommendations—gathered partly from stakeholder workshops (Krebs et al., 2023a; Krebs et al., 2025)—for funding bodies to begin tackling this issue.

- *Provide animal methods bias mitigation training for peer reviewers, review staff, and editors*: A critical step in mitigating animal methods bias is mandatory bias training that specifically addresses reviewers’ preference for animal-based methods. Such training is essential for both grant and publication review processes, helping reviewers recognize, understand, and manage their bias toward animal methods and helping review staff and editors identify it and intervene when necessary.
- *Integrate NAMs experts in review groups*: Animal methods bias often stems from a lack of knowledge or experience with human-based approaches. Including appropriate expertise in funding and publishing review is crucial to ensuring that human-based research is evaluated fairly and adequately.
- *Inform reviewers, review staff, and editors about the value of human-based methods:* For reviewers who are not experts in these methods, simple educational and awareness campaigns about nonanimal approaches and their value in translational success will be crucial for mitigating bias against them during review. Incorporating information gathered from systematic reviews of preclinical research can help improve awareness of methodological limitations and overall research quality, Menon et al. (2021) potentially also shifting perspectives of biased reviewers. E-learning platforms, such as the Education and Training Platform in Laboratory Animal Science, EU-52 from ETPLAS (2025) may be a good option for disseminating this information.
- *Facilitate the investigation of animal methods bias:* Understanding the full extent of animal methods bias and its impacts on funding and publishing rates of animal- and nonanimal-based studies is crucial for addressing it appropriately and holistically. Assessments of reviewer comments for evidence of bias toward animal methods should be conducted by funders and publishers or facilitated to be conducted by external meta-researchers and social scientists. Explicitly naming “animal methods bias” in policies and other materials will further support broader investigations and promote a standardized terminology to facilitate interdisciplinary dialogue, align mitigation frameworks, and promote harmonization across funding and publishing domains.
- *Develop policies and infrastructure to support NAMs*: Greater institutional support for NAMs will drive wider acceptance over time. This includes investing in NAMs training, establishing research hubs and core facilities, and creating opportunities for the development, validation, and implementation of NAMs.

While there have been exciting pushes in the US and UK toward mitigating animal methods bias, global commitment and cooperation will be required to fully remove it as a barrier against goals of improving research translation and reducing animal use. The Coalition to Illuminate and Address Animal Methods Bias developed an advocacy toolkit for researchers and advocates in other global regions to engage with local funding bodies to promote the fair review of NAMs and other nonanimal methods (Coalition to Illuminate and Address Animal Methods Bias (COLAAB), 2025a; Coalition to Illuminate and Address Animal Methods Bias (COLAAB), 2025b).

## 6 Discussion

Despite calls around the world to reduce animal use, some skeptics are resistant to these efforts (Lloyd, 2025). This resistance feeds into challenges faced by NAMs users when obtaining grant funding and publishing their studies (Herron et al., 2025). It is evident that animal methods bias pervades the scientific process, affecting peer review and slowing progress in the uptake of nonanimal, human-based approaches.

While several studies have been conducted investigating the impacts of animal methods bias in publishing and funding, we still need a better understanding of its impacts in other areas, such as conference abstract reviews, hiring and tenure evaluation, or the ways scientific developments are reported in the media. More research into the prevalence of animal methods bias around the world and how it manifests across different global regions is also needed to better implement policies attuned to specific research funding, infrastructure, legislative, and regulatory contexts.

While roadmaps from the US and UK have outlined plans to reduce animal use and address animal methods bias, the success of these implementations remains to be seen, further measures will likely be required, and other governing bodies must follow suit. Animal methods bias is not a peripheral issue—it affects every stage of biomedical research where methods are assessed. Addressing it is critical, not only to improve translation and bring more effective treatments to the clinic, but also to respond to the growing international call to end reliance on animal experimentation.

## Data Availability

The original contributions presented in the study are included in the article/supplementary material, further inquiries can be directed to the corresponding author.
